# Enteroviruses in Respiratory Samples from Paediatric Patients of a Tertiary Care Hospital in Germany

**DOI:** 10.3390/v13050882

**Published:** 2021-05-11

**Authors:** Susanne Baertl, Corinna Pietsch, Melanie Maier, Mario Hönemann, Sandra Bergs, Uwe G. Liebert

**Affiliations:** Institute of Medical Microbiology and Virology, Leipzig University Hospital, 04103 Leipzig, Germany; susanne.baertl@icloud.com (S.B.); melmai@medizin.uni-leipzig.de (M.M.); HoenemannM@medizin.uni-leipzig.de (M.H.); sandra.bergs@medizin.uni-leipzig.de (S.B.); liebert@medizin.uni-leipzig.de (U.G.L.)

**Keywords:** enteroviruses, enterovirus EV-D68, respiratory infections, molecular epidemiology, paediatric patients

## Abstract

Enteroviruses are associated with various diseases accompanied by rare but severe complications. In recent years, outbreaks of enterovirus D68 and enterovirus A71 associated with severe respiratory infections and neurological complications have been reported worldwide. Since information on molecular epidemiology in respiratory samples is still limited, the genetic diversity of enteroviruses was retrospectively analysed over a 4-year period (2013–2016) in respiratory samples from paediatric patients. Partial viral major capsid protein gene (VP1) sequences were determined for genotyping. Enteroviruses were detected in 255 (6.1%) of 4187 specimens. Phylogenetic analyses of 233 (91.4%) strains revealed 25 different genotypes distributed to *Enterovirus A* (39.1%), *Enterovirus B* (34.3%), and *Enterovirus D* (26.6%). The most frequently detected genotypes were enterovirus D68 (26.6%), coxsackievirus A6 (15.9%), and enterovirus A71 (7.3%). Enterovirus D68 detections were associated with lower respiratory tract infections and increased oxygen demand. Meningitis/encephalitis and other neurological symptoms were related to enterovirus A71, while coxsackievirus A6 was associated with upper respiratory diseases. Prematurity turned out as a potential risk factor for increased oxygen demand during enterovirus infections. The detailed analysis of epidemiological and clinical data contributes to the non-polio enterovirus surveillance in Europe and showed high and rapidly changing genetic diversity of circulating enteroviruses, including different enterovirus D68 variants.

## 1. Introduction

Human enteroviruses (EVs), belonging to the *Picornaviridae* family, are small single-stranded, non-enveloped RNA viruses. More than 100 human pathogenic genotypes are classified into the four species *Enterovirus A* to *Enterovirus D* [1]. These species include polioviruses, coxsackieviruses (CV), echoviruses (E), EV-A71, and EV-D68. Additionally, three rhinovirus genotypes are allocated to the genus *Enterovirus* [2,3].

Targeting the highly conserved 5′untranslated region (UTR), real-time reverse-transcription PCR (RT-PCR) is the diagnostic method of choice for EV detection in clinical samples. For genotyping and molecular epidemiological purposes, partial sequencing of the major capsid protein (VP1) region is recommended [4,5].

EVs are commonly transmitted by faecal-oral or respiratory routes. Most of them are swallowed and bound to specific receptors on enterocytes, and they cross the intestinal mucosa to replicate in the gut-associated lymphatic tissue [6]. Via short-lived viraemia, other tissues can be affected [1,7]. However, certain EVs, including EV-D68 and *Enterovirus C* viruses, share biological properties with rhinoviruses, such as acid sensitivity and temperature lability, and therefore exhibit distinct respiratory tropism. These so-called respiratory enteroviruses replicate in the epithelial cells of the respiratory tract and are therefore found predominantly or exclusively in respiratory samples, while others are only occasionally detectable [6,8,9].

EVs show high genetic diversity and are associated with a broad spectrum of diseases. In nine out of 10 cases, EV infections occur without or with mild unspecific symptoms. Clinical manifestations range from malaise, febrile illness, hand, foot and mouth disease (HFMD), and mild upper respiratory tract infections to severe respiratory illnesses, such as obstructive bronchitis or pneumonia and neurological manifestations such as meningitis, encephalitis, or acute flaccid paralysis (AFP) [1,8,10]. Age, gender, pre-existing conditions, and the host immune status are supposed to influence clinical presentation and disease severity [8].

EVs account for more than 10 million infections per year and several thousand hospital admissions in the USA. Along with human rhinoviruses, EVs are among the most common causative agents of human disease and are a significant burden to the patients as well as to the health care system [11,12,13]. From 2008 onwards, outbreaks of severe respiratory infections and cases of AFP associated with EV-D68 infections were reported in the USA, Canada, and in Europe [13,14]. The largest outbreak of EV-D68, which occurred in Northern America in 2014, led to severe respiratory and neurological diseases, mainly in children, and caused considerable hospitalisation rates. Especially children with underlying disease, such as asthma, were affected [13,15,16].

In Asia, EV-A71 infections were responsible for large outbreaks of HFMD, complicated by brainstem encephalitis and severe cardiorespiratory symptoms. In Europe and in the USA, HFMD is a self-limiting and sporadic disease, which is mainly associated with CVA16 and CVA6, more rarely with EV-A71 [5,12,16].

The global emergence of EV-D68 and EV-A71 shows the importance of strengthening and continuing epidemiological research on respiratory EVs [5,10,16,17]. Therefore, the present study aims to investigate the molecular epidemiology and clinical presentations of EV infections in infants and children from a German medical university centre over a 4-year period.

## 2. Materials and Methods

### 2.1. Patients and Samples

Between March 2013 and December 2016, 4187 respiratory specimens were collected from individual infants and children (<16 years of age) at the University Hospital of Leipzig. Respiratory samples consisted of nasal secretions, naso- or oropharyngeal swabs, or tracheal secretions. Patients’ characteristics and clinical data were extracted from the medical charts for EV positive patients. Clinical phenotypes were identified based on the diagnoses and information in the patients’ discharge records provided by the attending paediatrician and categorised as followed: (1) upper respiratory tract infections (e.g., sinusitis, pharyngitis), (2) obstructive bronchitis, (3) pneumonia, (4) other respiratory symptoms (e.g., infant respiratory distress syndrome), (5) gastrointestinal symptoms (e.g., diarrhoea, vomiting), (6) HFMD and/or exanthema, (7) meningitis or encephalitis, (8) other neurological symptoms (e.g., disorientation, somnolence), (9) seizures, (10) fever, and (11) other clinical manifestations. Considered pre-existing conditions were prematurity (<37 gestational weeks), asthma, epilepsy, and immunocompromised status. Disease severity was measured by admission to intensive care unit (ICU), oxygen demand, and duration of hospital stay.

The Leipzig University Ethics Committee approved the study design (no. Az 301/16-ek).

### 2.2. Viral Detection and Typing

Total nucleic acid (NA) was extracted from respiratory samples using the DNA and Viral NA Small Volume Kit and the MagNA Pure 96 instrument (Roche, Mannheim, Germany) according to the manufacturer’s instructions. Extracted NA were tested with a commercially available test (NxTAG RPP, Luminex Corporation, Austin, TX, USA) for respiratory viruses including EVs and rhinoviruses. According to the manufacturer, the limit of detection for rhinovirus/enterovirus in the Luminex assay was determined to be 1.1 × 10^3^ copies/mL sample.

The presence of EV RNA was confirmed by a RT-qPCR targeting the highly conserved 5′UTR [18] with a cycle threshold value ≤ 40. In case of EV-positive results, partial VP1 viral capsid gene region was amplified using primers AN88 and AN89 [4,18]. DNA amplicons were gel-purified (Wizard SV Gel and PCR Clean-Up System, Promega Corporation, Madison, WI, USA) and sequenced using BigDyeTerminator v1.1 Cycle Sequencing Kit on a 3500 Genetic Analyzer (both, Thermo Fisher Scientific, Applied Biosystem, Waltham, MA, USA). Sequences were analysed (Geneious Prime Biomatters, Auckland, New Zealand) and genotyped (Enterovirus Genotyping Tool Version 0.1, National Institute for Public Health and the Environment, Bilthoven, The Netherlands) [19]. Identified sequences were submitted to GenBank (accession no. MW731790 to MW732022).

### 2.3. Statistics

Frequencies were expressed as numbers and percentages. Continuous parameters were presented as median values and compared by ranks using the Kruskal–Wallis Test and Bonferroni post hoc test for continuous data across multiple groups and in case of pairwise comparison by Wilcoxon-Mann-Whitney U-test. Chi-square test and Fisher’s exact test, if required, were used for comparison of categorical variables. For comparison of more than two groups, a pairwise Chi-square test or Fisher’s exact test was performed in a second step. The association between categorical variables and severity was analysed using univariate logistic regression. Therefore, continuous variables were dichotomised by median values and treated as binary variables. The presented significance level was set at *p* ≤ 0.05. Descriptive and statistical data analysis was performed using the IBM SPSS Statistics software (version 24.0, IBM Corp, Armonk, NY, USA).

## 3. Results

### 3.1. Detection of Enteroviruses and Molecular Characterisation

Samples were obtained from 2390 (57.1%) male and 1795 (42.9%) female patients. In two cases, no gender was indicated.

In total, 255 (6.1%) samples were confirmed positive for EV RNA by RT-qPCR. Positive-tested respiratory specimens consisted of 241 (94.5%) naso- and/or oropharyngeal swabs, 10 (3.9%) nasal secretions, and 4 (1.6%) tracheal secretions.

**Figure 1:**
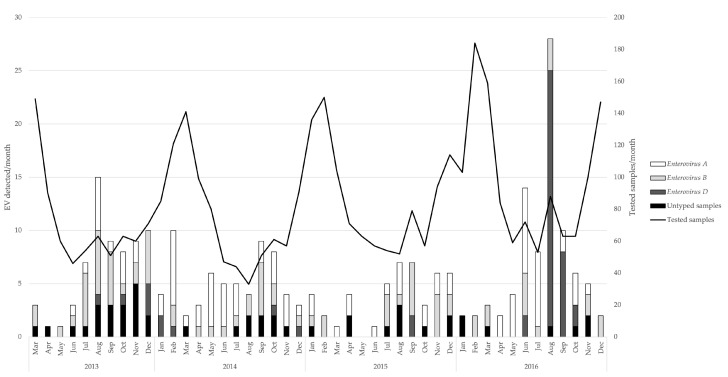
Enterovirus detection (*n* = 255) stratified by species A, B, D, and not typeable, as well as overall tested samples by month, during 2013–2016. Left axis showing absolute numbers of *Enterovirus A*, *Enterovirus B,* and *Enterovirus D* viruses, as well as of untyped samples. Right axis showing the absolute number of tested samples.

The positivity rate ranged from 4.3% (44/1031) in 2015 to 7.7% (90/1175) in 2016, while it was at 5.9% (63/1070) in 2013 and at 6.4% (58/911) in 2014. Monthly detection rates showed seasonal fluctuations with an increased prevalence in summer and early autumn (May–September) (Figure 1).

Out of 255 EV-positive samples, 233 (91.4 %) could be genotyped (Table 1). Of those, 91 (39.1%) were assigned to *Enterovirus A*, 80 (34.3%) were assigned to *Enterovirus B*, and 62 (26.6%) were assigned to *Enterovirus D*, which comprised 7, 17, and 1 genotype(s), respectively (Table 1). All in all, EV-D68 was detected most frequently, followed by CVA6 and EV-A71. Within the EV-D68 genotype, clade B3, B2, B1, and A2 viruses occurred, exhibiting a shift from non-B3 towards B3-clade EV-D68 viruses during 2015.

### 3.2. Patients’ Characteristics and Clinical Symptoms

Patients’ characteristics and clinical symptoms are presented in Table 2. The detection rate in <1-year-old and >5–16-year-old patients was lower (3.6% each) than in the 1 or 2–5-year-old patients (7.3% to 8.0%, respectively). The age distribution of positively tested patients ranged from 8 days to 15 years, resulting in a median age of 22 months. The detected EV species showed significant differences in median age. Precisely, *Enterovirus A* viruses were found more often in younger patients than *Enterovirus B* viruses (*p* = 0.032) or *Enterovirus D* viruses (*p* < 0.001). The youngest median age was observed in patients affected by EV-A71 (13 months) and CVA6 (15 months). The median length of hospital stay was 3 days. The longest treatment period, 59 days, was observed in a premature infant infected with EV-A71. Admission to ICU was required in 23 (10.4%) cases, with a median length of stay of 6 days, ranging from 1 to 38 days. The median duration of ventilation was 3 days. Oxygen demand was indicated in 45 (20.3%) patients and was significantly associated with the presence of EV-D68 (Table 2).

Clinical manifestations included mainly upper respiratory tract symptoms (*n* = 105; 45.7%), fever (*n* = 64; 27.8%), obstructive bronchitis (*n* = 61; 26.5%), gastrointestinal symptoms (*n* = 49; 21.3%), and HFMD or exanthema (*n* = 30; 13.0%). Upper respiratory tract infections were frequently associated with *Enterovirus A* viruses, with CVA6 (22/105; 21.0%) being the most common detected type. Furthermore, CVA6 was associated with gastroenteritis (15/49; 30.6%) and HFMD or exanthema (19/30; 63.3%). Enterovirus D68 was the most common EV genotype in patients with obstructive bronchitis (42/61; 68.9%) and pneumonia (9/19; 47.4%). Neurological manifestations occurred in 20 cases. Of those with meningitis or encephalitis (*n* = 10), four were positive for EV-A71 and one for EV-D68. The EV-D68 patient showed acute flaccid paralysis of the lower limbs, brainstem encephalitis, myelitis, sixth nerve palsy, and ataxia. In this patient, EV-D68 was the only detected pathogen. After 12 days duration of hospital stay, the patient was discharged with persistent weakness of both legs.

### 3.3. Pre-Existing Conditions

Forty-nine (21.3%) EV positive patients had pre-existing conditions. Among these, 22 (9.6%) patients were premature infants, 12 (5.2%) suffered from asthma, 11 (4.8%) suffered from epilepsy, and 6 (2.6%) were immunocompromised. Within the subgroup of asthma patients, EV-D68 was the most frequently detected genotype (9/12).

Prematurity and EV-D68 detections were associated with a higher risk for oxygen demand, but there was no difference in ICU admission or duration of stay. Neither age, nor gender, nor asthma were significant determinants of disease severity. The presence of epilepsy or immunosuppression as potential predictors for severity was also not statistically significant (Table 3).

## 4. Discussion

Respiratory EVs still seem to be an underestimated agent, both in terms of morbidity especially in children <5 years and in terms of socioeconomic burdens [6,16]. Therefore, the presented study aimed to investigate the epidemiology and clinical spectrum of EVs in respiratory samples from paediatric patients in a German university medical centre.

In our study population, the overall frequency of EV infections in respiratory specimens was 6.1%, although it reached up to 33.0% during the summer months. Cabrerizo et al. described a frequency of 6.5% in samples from infants and children; however, only 9% of the samples originated from the respiratory tract [20]. Lower detection rates (2%) were reported in the Netherlands, which was probably because of the selective inclusion of patients with respiratory symptoms [21]. Nevertheless, Andres et al. showed a similar rate of 7% by exclusively testing respiratory specimens from children. As reported in other studies on the outbreaks in 2014 and 2016 [20,22,23,24], a remarkable increase in EV detections and positivity rates was observed for 2014 and especially for 2016. These high prevalence seasons were closely linked to high detection rates of EV-D68 in 2016 and, albeit to a lower extent, in the winter months of 2013/2014. In line with similar upsurges of EV-D68 reported by European countries and the USA [23,24,25,26,27,28], a biennial periodicity pattern of EV-D68 spreading is supported by the presented results.

In addition, detection rates were also comparatively high in 2013. However, this should be interpreted cautiously, as sampling for genotype analysis started in March 2013. This may have led to higher results due to missing data from January and February usually characterised by lower EV incidences. Furthermore, detection rates could be biased due to increased awareness and sampling after reported outbreaks. However, this seems unlikely, since the total number of samples received per month did not grossly differ for 2013, 2014, and 2015, compared to 2016. Finally, the incidence of EV may have been influenced by nosocomial outbreaks, as samples were collected mainly from hospitalised patients.

Regarding seasonality, higher detection rates in the summer months are in line with the typical and well-studied seasonal circulations of EVs with peaks in summer and early autumn [16,29,30]. However, EVs circulated throughout the year and a relatively higher incidence during the winter season as reported by Benschop et al. [29] were observed in winter 2013/2014. As a result of the varying seasonality, the present data support that consideration of enteroviruses as a differential diagnosis should not be limited to specific seasons.

In comparison to other studies [10,20,21], a high rate (91.4%) of successfully genotyped strains could be achieved. With 25 different genotypes identified, genetic diversity was similar or even higher compared to studies that included non-respiratory samples in their investigations [20,30]. The highest prevalence was revealed for *Enterovirus A* viruses; the most prevalent genotypes in our study population were EV-D68, CVA6, and EV-A71.

In total, 62 EV-D68 positive samples were identified. Due to its biological properties shared with rhinoviruses, such as acid lability and sensitivity to high temperature (>35 °C), this genotype shows a high affinity for the respiratory tract [5,6]. Therefore, EV-D68 is almost exclusively detectable in respiratory samples, whereas the other genotypes identified in the present report are occasionally found in this sample type [5,6,8,9].

Clade B viruses were detected in all EV-D68 positive samples except one EV-D68 of clade A. In 2016, most of the sequences clustered to clade B3, whereby in 2014, clade B1 and B2 viruses were predominantly circulating. This shift from B1 and B2 clades and less frequently detected A1 and A2 clades in 2014 to clade B3 in 2016 was also reported during and after the emerging North American outbreak as well as by pan-European surveillance data [17,22,23,26,31].

Clinical data could be obtained in most of the patients included in this study. EVs were mainly detected in young children under 5 years of age. The data regarding patients’ age are in line with previous reports [1,16,32]. The high risk of EV infections might be explained by an increased vulnerability to infection of infants. Conceivably, the immature immune system and smaller airways lead to more severe symptoms and higher hospitalisation rates [27]. *Enterovirus A* viruses were found in younger patients than *Enterovirus B* and *Enterovirus D* viruses, while CVA6 and EV-A71 affected the youngest infants. Even though 44.4% of EV-A71 infections occurred in neonates, no specific association to neonatal infections was shown, which is in contrast to other reports that indicated EV-A71 being more prevalent in neonates than in children aged >1 month [20,33].

In addition to its well-described clinical manifestations (e.g., HFMD, gastroenteritis), CVA6 was additionally associated with mild upper respiratory tract infections in our study cohort. It is well known that owed to the wide spectrum of clinical manifestations caused by EVs, clinical phenotypes are not restricted to specific genotypes [1,8].

In recent years, EV-D68 infections were associated with acute respiratory infections and neurological complications such as AFP [17,22,25,26,31]. Most of these investigations were based on the analysis of samples from hospitalised patients, and therefore, data on associated symptoms and pre-existing conditions are potentially biased towards higher severity. Surveillance data from outpatients in the Netherlands, Canada, and Germany indicate more mild courses of influenza-like illness in EV-D68 infections [15,34,35]. Therefore, the present study design may reflect a tip-of-the iceberg phenomenon in which more severe cases and the proportion of pre-existing conditions are overrepresented but is also very sensitive to detect severe complications of EV infections.

Two EV-D68-positive patients presenting with neurological symptoms were identified during the study period. Of them, one child showed symptoms of AFP such as acute flaccid paralysis of the lower limbs, brainstem encephalitis, myelitis, sixth nerve palsy, and ataxia. EV-D68 was the only detected pathogen in this patient. As described by Knoester et al. [25], the diagnosis of AFP can be complicated by subtle radiological and electromyography results. Nevertheless, the association between EV-D68 and AFP has become accepted in recent years. Even though causality has not been proven so far, reports and an animal model in which EV-D68 induced paralysis in neonatal mice are contributing to increasing evidence [36,37,38].

Consistent with previous studies [13,23,28,39], EV-D68 was associated with severe obstructive bronchitis and pneumonia, which are characterised by higher rates of oxygen demand. Additionally, premature infants also had higher rates of oxygen demand than those without.

By contrast, *Enterovirus A* and *Enterovirus B* viruses were significantly more frequently detected in upper respiratory tract infections, which was also described by González-Sanz et al. [23]. Rates of ICU admissions did not differ significantly between species, although previous studies reported higher rates in patients infected with EV-D68 [26,28].

In our study cohort of primarily hospitalised patients, 21.3% of the infants showed pre-existing conditions. These results could be limited by increased collection of samples in chronically ill infants, or EV infections in those without pre-existing conditions more often stayed sub-clinical. Nevertheless, there is some evidence that pre-existing conditions in general have an impact on hospitalisation rate and disease severity in EV infection [13,17]. Asthma was not a significant risk factor for higher disease severity in the present study cohort. Yet, the present infants with asthma as an underlying disease were more often affected by EV-D68 than by other genotypes. However, due to the small number of asthma patients in the present study, this finding can only be regarded as preliminary.

EV-A71 known as a major agent for HFMD and encephalitis in the Asia Pacific region was recently associated with severe respiratory infections mainly in young children [6,14]. Furthermore, EV-A71 was responsible for large HFMD outbreaks accompanied by severe neurological and cardio-pulmonic complications in remarkable numbers in Taiwan (1.5 million cases), Malaysia (Sarawak, 2628 cases), and China (490,000 cases), most frequently affecting children [12,40]. These outbreaks in the Asian Pacific region were mainly caused by strains of subgenogroups B3, B4, C3, C4, and C5 [6,41]. In contrast, EV-A71 sublineages C1 and C2 circulated at a low level in Africa, USA, and Europe, causing only small outbreaks or occasional cases [12,41]. Likewise, genotypes of EV-A71 detected in the present study clustered mainly in subgenogroup C1 and to a small extent in subgenogroup C2.

Among the patients admitted with EV-A71, no cases of HFMD were noticed, which may be due to preselection for respiratory samples. Although EV-A71 was predominantly associated with meningitis or encephalitis and with other neurologic symptoms in this study, the detection of EVs in cerebrospinal fluid was not evaluated, so no definite etiologic association can be drawn between neurologic symptoms and the presence of EV in the respiratory tract.

Nevertheless, there seems to be a mismatch between the ongoing threat of EV-A71 in the Asia Pacific region and the low infection rates in the Western world. Epidemiological parameters such as overcrowding, poor hygiene, and climate conditions could foster virus transmission and explain the high morbidity rates in the Asia Pacific region. In addition, the different virulence of the EV-A71 genogroups and host susceptibility of the population may be possible explanations [41]. In Australia and Malaysia, higher complication rates were associated with EV-A71 subtypes C2 and B5 and more frequent expression of HLA haplotypes in the Asian population [12].

Except for a vaccine for EV-A71 available in China [42], there are no further prophylactic interventions available for non-polio EVs. In recent years, efforts have been made to establish effective therapeutic strategies especially for patients with severe neurological complications. Possible therapy options consist of various antiviral drugs such as pleconaril, intravenous immunoglobulin therapy, or corticosteroids, which show inconsistent results [13,16,43].

## 5. Conclusions

This study reports on the clinical spectrum and epidemiology of EVs in respiratory samples from paediatric patients, treated at a tertiary care university hospital in Germany. Even though larger epidemiologic studies are needed, the present report on circulation patterns and severe respiratory infections are consistent with other studies and highlight EVs as an ongoing and observable menace. Recently established pan-European or national networks, such as the European Non-polio Enterovirus Network (ENPEN) [5] or VIRO-TypeNed [29,43], have improved the understanding of circulation patterns by sharing data on emerging EVs, disease outbreaks and the harmonisation of EV diagnostic methods. Yet, they are still based on voluntary reporting and testing [5,44].

Enterovirus surveillance programs will not sufficiently recognise EV-D68 and other important enteroviruses — unless they are extended to include the routine screening of respiratory samples and subsequent characterisation of enteroviruses genotypes. In addition to that, future objectives will be to raise awareness of EVs as causative agents of severe respiratory diseases in clinical practice. Moreover, the recognition of EV-associated diseases allows us to better assess the burden of EV disease, to monitor the occurrence of new strains, and is also necessary for the possible implementation of vaccination programmes and therapy strategies.

With a detailed analysis of epidemiological and clinical data of EV infections in children focussing on respiratory samples, this study provides a further important contribution to non-polio EV surveillance in Europe, particularly in Germany, and emphasises that EV detection without characterisation is not enough to recognise ever-changing emerging genotypes and future outbreaks.

## Figures and Tables

**Table 1:** Enteroviruses detected from March 2013 to December 2016 (*n* = 255).

Species	Genotypes	2013	2014	2015	2016	Total
*Enterovirus A*	CVA2	4	2	-	2	8
	CVA4	-	3	3	2	8
	CVA5	1	2	2	-	5
	CVA6	3	18	9	7	37
	CVA10	-	4	-	5	9
	CVA16	2	4	-	1	7
	EV-A71, Subgenogroup C1	1	-	2	10	13
	EV-A71, Subgenogroup C2	2	-	1	1	4
*Enterovirus B*	CVA9	1	2	-	1	4
	CVB1	2	-	1	-	3
	CVB2	2	2	3	1	8
	CVB3	5	2	-	2	9
	CVB4	5	-	2	1	8
	CVB5	1	2	2	4	9
	E3	2	2	-	-	4
	E5	-	-	-	1	1
	E6	-	1	-	-	1
	E7	-	-	5	2	7
	E9	1	2	-	-	3
	E11	1	-	-	-	1
	E18	-	2	3	2	7
	E20	-	-	1	-	1
	E25	1	-	-	1	2
	E27	-	-	1	-	1
	E30	9	-	-	2	11
*Enterovirus D*	EV-D68, Clade A2	-	1	-	-	1
	EV-D68, Clade B1	1	3	-	-	4
	EV-D68, Clade B2	15	4	-	1	20
	EV-D68, Clade B3	-	-	2	35	37
Untyped		4	2	7	9	22
	Total	63	58	44	90	255

EV: enterovirus; CV: coxsackievirus; E: echovirus.

**Table 2:** Distribution of enterovirus species according to clinical phenotypes, March 2013 to December 2016 (*n* = 230 ^1^).

	*Enterovirus A*	*Enterovirus B*	*Enterovirus D*	Total	*p* *
Number	90	78	62	230	
**Characteristics**					
Median age, months	19 ^b,d^	24	32	22	**<0.001**
Median duration of hospital stay, days	3	3	3.5	3	0.155
Median duration of ICU stay, days	5.5	4	7	6	0.757
Oxygen demand, *n* (%)	8 (17.8%)	11 (24.4%)	26 (57.8%) ^a,b^	45	**<0.001**
**Clinical Symptoms**					
Upper respiratory tract, *n* (%)	51 (48.6%) ^d^	43 (41.0%) ^d^	11 (10.5%)	105	**<0.001**
Obstructive bronchitis, *n* (%)	8 (13.1%)	11 (18.0%)	42 (68.9%) ^a,b^	61	**<0.001**
Pneumonia, *n* (%)	2 (10.5%)	8 (42.1%) ^a^	9 (47.4%) ^a^	19	**0.013**
Other respiratory symptoms, *n* (%)	3 (42.9%)	1 (14.3%)	3 (42.9%)	7	0.439
Gastrointestinal symptoms, *n* (%)	29 (59.2%) ^b,d^	13 (26.5%)	7 (14.3%)	49	**0.004**
HFMD/exanthema, *n* (%)	23 (76.7%) ^b,d^	7 (23.3%) ^d^	0	30	**<0.001**
Meningitis/encephalitis, *n* (%)	5 (50.0%)	4 (40.0%)	1 (10.0%)	10	0.510
Other neurological symptoms, *n* (%)	6 (60.0%)	3 (30.0%)	1 (10.0%)	10	0.327
Seizures, *n* (%)	22 (53.7%) ^d^	15 (36.6%) ^d^	4 (9.8%)	41	**0.011**
Fever, *n* (%)	33 (51.6%) ^d^	28 (43.8%) ^d^	3 (4.7%)	64	**<0.001**
Others, *n* (%)	16 (55.2%) ^d^	13 (44.8%) ^d^	0	29	**0.002**

*p*: *p*-value; ICU: intensive care unit; HFMD: hand, foot, and mouth disease. ^1^ Clinical data of three EV positive patients could not be obtained; * Chi-square or Fisher’s exact test for categorical variables and Kruskal–Wallis test (median) for continuous variables; *p*-values numbers marked in bold indicate significance (*p* < 0.05). Bonferroni post hoc test (median) or pairwise Chi-square test/Fisher’s exact test (categorical variables): *p* < 0.05 ^a^ compared to *Enterovirus A*; ^b^ compared to *Enterovirus B*; ^d^ compared to *Enterovirus D.* Clinical phenotypes are categorized into patients’ characteristics and clinical symptoms (left column, bold).

**Table 3:** Analysis of potential risk factors for disease severity in children infected with EVs, March 2013 to December 2016 (*n* = 222 ^1^).

Characteristics	ICU Admission				Oxygen Demand				Duration of Hospital Stay ^2^			
	Yes	No	*p*	OR (95% CI)	Yes	No	*p*	OR (95% CI)	>3 Days	≤3 Days	*p*	OR (95% CI)
**Sex**												
Male	15	121	0.681	1.21 (0.49–2.99)	23	113	0.120	0.59 (0.31–1.15)	57	79	0.601	1.16 (0.67–2.01)
Female	8	78			22	64			33	53		
**Age ^2^**												
≤22 months	14	100	0.337	1.54 (0.64–3.72)	27	87	0.195	1.55 (0.80–3.02)	50	64	0.301	1.33 (0.78–2.27)
>23 months	9	99			18	90			40	68		
**Prematurity**												
Yes	3	17	0.479	1.61 (0.43–5.96)	9	11	**0.006** *	3.77 (1.46–9.77)	12	8	0.069	2.39 (0.93–6.10)
No	20	182			36	166			78	124		
**Asthma**												
Yes	2	10	0.467	1.80 (0.37–8.77)	4	8	0.256	2.06 (0.59–7.18)	5	7	0.935	1.05 (0.32–3.42)
No	21	189			41	169			85	125		
**Epilepsy**												
Yes	2	9	0.391	2.01 (0.41–9.93)	3	8	0.556	1.51 (0.38–5.93)	7	4	0.122	2.70 (0.77–9.51)
No	21	190			42	169			83	128		
**Immunocompromised status**												
Yes	1	5	0.612	1.76 (0.20–15.7)	0	6	NA	NA	4	2	0.207	3.02 (0.54–16.87)
No	22	194			45	171			86	130		
**EV–D68 infection**												
Yes	9	45	0.086	2.20 (0.89–5.42)	26	28	**<0.001**	7.28 (3.56–14.90)	27	27	0.105	1.67 (0.90–3.09)
No	14	154			19	149			63	105		

CI: confidence interval; OR: odds ratio; EV: enterovirus; ICU: intensive care unit; NA: not applicable because of the small number of reports; ***p***: *p*-value. ^1^ Complete data were not available for eleven patients. ^2^ Dichotomised according to median value. Median duration of hospital stay was three days. Median patient age was 22 months. * *p*-values numbers marked in bold indicate significance (*p* < 0.05). Sex, age, prematurity, asthma, epilepsy, immunocompromised status and EV-D68 infection were considered as potential risk factors for disease severity and are marked in bold (left column).

## Data Availability

Identified sequences were submitted to GenBank (accession No. MW731790 to MW732022).

## References

[1] Oberste M.S., Gerber S.I., Kaslow R.A., Stanberry L.R., Le Duc J.W. (2014). Enteroviruses and Parechoviruses: Echoviruses, Coxsackieviruses, and Others. Viral Infections of Humans.

[2] Picornavirus Home. https://www.picornaviridae.com.

[3] Pallansch M., Roos R., Knipe D.M., Howley P.M. (2006). Enteroviruses. Fields Virology.

[4] Nix W.A., Oberste M.S., Pallansch M.A. (2006). Sensitive, Seminested PCR Amplification of VP1 Sequences for Direct Identification of All Enterovirus Serotypes from Original Clinical Specimens. J. Clin. Microbiol..

[5] Harvala H., Broberg E., Benschop K., Berginc N., Ladhani S., Susi P., Christiansen C., McKenna J., Allen D., Makiello P. (2018). Recommendations for enterovirus diagnostics and characterisation within and beyond Europe. J. Clin. Virol..

[6] Royston L., Tapparel C. (2016). Rhinoviruses and Respiratory Enteroviruses: Not as Simple as ABC. Viruses.

[7] De Crom S.C.M., Rossen J.W.A., Van Furth A.M., Obihara C.C. (2016). Enterovirus and parechovirus infection in children: A brief overview. Eur. J. Nucl. Med. Mol. Imaging.

[8] Tapparel C., Siegrist F., Petty T.J., Kaiser L. (2013). Picornavirus and enterovirus diversity with associated human diseases. Infect. Genet. Evol..

[9] Tapparel C., Junier T., Gerlach D., Van Belle S., Turin L., Cordey S., Mühlemann K., Regamey N., Aubert J.-D., Soccal P.M. (2009). New Respiratory Enterovirus and Recombinant Rhinoviruses among Circulating Picornaviruses. Emerg. Infect. Dis..

[10] Andrés C., Vila J., Gimferrer L., Piñana M., Esperalba J., Codina M.G., Barnés M., Martín M.C., Fuentes F., Rubio S. (2019). Surveillance of enteroviruses from paediatric patients attended at a tertiary hospital in Catalonia from 2014 to 2017. J. Clin. Virol..

[11] Fine J., Bray-Aschenbrenner A., Williams H., Buchanan P., Werner J. (2018). The Resource Burden of Infections with Rhinovirus/Enterovirus, Influenza, and Respiratory Syncytial Virus in Children. Clin. Pediatr..

[12] Solomon T., Lewthwaite P., Perera D., Cardosa M.J., McMinn P., Ooi M.H. (2010). Virology, epidemiology, pathogenesis, and control of enterovirus. Lancet Infect. Dis..

[13] Holm-Hansen C.C., Midgley S.E., Fischer T.K. (2016). Global emergence of enterovirus D68: A systematic review. Lancet Infect. Dis..

[14] Holm-Hansen C.C., Midgley S.E., Schjørring S., Fischer T.K. (2017). The importance of enterovirus surveillance in a Post-polio world. Clin. Microbiol. Infect..

[15] Meijer A., Benschop K.S., Donker G., Van Der Avoort H.G. (2014). Continued seasonal circulation of enterovirus D68 in the Netherlands, 2011. Eurosurveillance.

[16] Rapid Risk Assessment—Enterovirus Detections Associated with Severe Neurological Symptoms in Children and Adults in European Countries, 8 August 2016. https://www.ecdc.europa.eu/sites/portal/files/media/en/publications/Publications/01-08-2016-RRA-Enterovirus%2071-Spain,%20France,%20Netherlands.pdf.

[17] Montes M., Oñate E., Muguruza A., Tamayo E., Carrera I.M., Iturzaeta A., Cilla G. (2019). Enterovirus D68 Causing Acute Respiratory Infection: Clinical Characteristics and Differences with Acute Respiratory Infections Associated With Enterovirus Non-D. Pediatr. Infect. Dis. J..

[18] Gelaw A., Pietsch C., Tigabu Z., Liebert U.G. (2020). Genotyping of enteroviruses and human parechoviruses highlights their diversity in Northwest Ethiopia. J. Med. Virol..

[19] Kroneman A., Vennema H., Deforche K., Avoort H., Peñaranda S., Oberste M., Vinjé J., Koopmans M. (2011). An automated genotyping tool for enteroviruses and noroviruses. J. Clin. Virol..

[20] Cabrerizo M., Díaz-Cerio M., Muñoz-Almagro C., Rabella N., Tarragó D., Romero M.P., Pena M.J., Calvo C., Rey-Cao S., Moreno-Docón A. (2017). Molecular epidemiology of enterovirus and parechovirus infections according to patient age over a 4-year period in Spain. J. Med. Virol..

[21] Poelman R., Schölvinck E.H., Borger R., Niesters H.G., Van Leer-Buter C. (2015). The emergence of enterovirus D68 in a Dutch University Medical Center and the necessity for routinely screening for respiratory viruses. J. Clin. Virol..

[22] Poelman R., Schuffenecker I., Van Leer-Buter C., Josset L., Niesters H.G., Lina B. (2015). European surveillance for enterovirus D68 during the emerging North-American outbreak in 2014. J. Clin. Virol..

[23] González-Sanz R., Taravillo I., Reina J., Navascués A., Moreno-Docón A., Aranzamendi M., Romero M.P., Del Cuerpo M., Pérez-González C., Pérez-Castro S. (2019). Enterovirus D68-associated respiratory and neurological illness in Spain, 2014. Emerg. Microbes Infect..

[24] Piralla A., Principi N., Ruggiero L., Girello A., Giardina F., De Sando E., Caimmi S., Bianchini S., Marseglia G.L., Lunghi G. (2018). Enterovirus-D68 (EV-D68) in pediatric patients with respiratory infection: The circulation of a new B3 clade in Italy. J. Clin. Virol..

[25] Knoester M., Schölvinck E.H., Poelman R., Smit S., Vermont C.L., Niesters H.G.M., Van Leer-Buter C.C. (2017). Upsurge of Enterovirus D68, the Netherlands. Emerg. Infect. Dis..

[26] Dyrdak R., Grabbe M., Hammas B., Ekwall J., Hansson K., Luthander J., Naucler P., Reinius H., Rotzén-Östlund M., Albert J. (2016). Outbreak of enterovirus D68 of the new B3 lineage in Stockholm, Sweden, August to September. Eurosurveillance.

[27] Kramer R., Sabatier M., Wirth T., Pichon M., Lina B., Schuffenecker I., Josset L. (2018). Molecular diversity and biennial circulation of enterovirus D68: A systematic screening study in Lyon, France, 2010 to 2016. Eurosurveillance.

[28] Piralla A., Girello A., Grignani M., Gozalo-Margüello M., Marchi A., Marseglia G., Baldanti F. (2013). Phylogenetic characterization of enterovirus 68 strains in patients with respiratory syndromes in Italy. J. Med. Virol..

[29] Benschop K.S.M., Rahamat-Langendoen J.C., Van Der Avoort H.G.A.M., Claas E.C.J., Pas S.D., Schuurman R., Verweij J.J., Wolthers K.C., Niesters H.G.M., Koopmans M.P.G. (2016). VIRO-TypeNed, systematic molecular surveillance of enteroviruses in the Netherlands between 2010 and 2014. Eurosurveillance.

[30] de Crom S., Rossen J., de Moor R., Veldkamp E., van Furth A., Obihara C. (2016). Prospective assessment of clinical symptoms associated with enterovirus and parechovirus genotypes in a multicenter study in Dutch children. J. Clin. Virol..

[31] Antona D., Kossorotoff M., Schuffenecker I., Mirand A., Leruez-Ville M., Bassi C., Aubart M., Moulin F., Lévy-Bruhl D., Henquell C. (2016). Severe paediatric conditions linked with EV-A71 and EV-D68, France, May to October. Eurosurveillance.

[32] Böttcher S., Prifert C., Weißbrich B., Adams O., Aldabbagh S., Eis-Hübinger A.M., Diedrich S. (2016). Detection of enterovirus D68 in patients hospitalised in three tertiary university hospitals in Germany, 2013 to 2014. Eurosurveillance.

[33] Khetsuriani N., LaMonte A., Oberste M.S., Pallansch M. (2006). Neonatal Enterovirus Infections Reported to the National Enterovirus Surveillance System in the United States, 1983–2003. Pediatr. Infect. Dis. J..

[34] Skowronski D.M., Chambers C., Sabaiduc S., Murti M., Gustafson R., Pollock S., Hoyano D., Rempel S., Allison S., De Serres G. (2015). Systematic community- and hospital-based surveillance for enterovirus-D68 in three Canadian provinces, August to December. Eurosurveillance.

[35] Reiche J., Böttcher S., Diedrich S., Buchholz U., Buda S., Haas W., Schweiger B., Wolff T. (2015). Low-level Circulation of Enterovirus D68–Associated Acute Respiratory Infections, Germany. Emerg. Infect. Dis..

[36] Hixon A.M., Yu G., Leser J.S., Yagi S., Clarke P., Chiu C.Y., Tyler K.L. (2017). A mouse model of paralytic myelitis caused by enterovirus D. PLoS Pathog..

[37] Dyda A., Stelzer-Braid S., Adam D., Chughtai A., MacIntyre C.R. (2018). The association between acute flaccid myelitis (AFM) and Enterovirus D68 (EV-D68)—What is the evidence for causation?. Eurosurveillance.

[38] Messacar K., Schreiner T.L., Maloney J., Wallace A., Ludke J., Oberste M.S., Nix W.A., Robinson C.C., Glodé M.P., Abzug M.J. (2015). A cluster of acute flaccid paralysis and cranial nerve dysfunction temporally associated with an outbreak of enterovirus D68 in children in Colorado, USA. Lancet.

[39] Bragstad K., Jakobsen K., Rojahn A.E., Skram M.K., Vainio K., Holberg-Petersen M., Hungnes O., Dudman S.G., Kran A.-M.B. (2014). High frequency of enterovirus D68 in children hospitalised with respiratory illness in Norway, autumn 2014. Influ. Other Respir. Viruses.

[40] Sabanathan S., Van Tan L., Thwaites L., Wills B., Qui P.T., Van Doorn H.R. (2014). Enterovirus 71 related severe hand, foot and mouth disease outbreaks in South-East Asia: Current situation and ongoing challenges. J. Epidemiol. Community Heal..

[41] Van Der Sanden S., Koopmans M., Uslu G., Van Der Avoort H., on behalf of the Dutch Working Group for Clinical Virology (2009). Epidemiology of Enterovirus 71 in The Netherlands, 1963 to 2008. J. Clin. Microbiol..

[42] Zhu F., Xu W., Xia J., Liang Z., Liu Y., Zhang X., Tan X., Wang L., Mao Q., Wu J. (2014). Efficacy, Safety, and Immunogenicity of an Enterovirus 71 Vaccine in China. N. Engl. J. Med..

[43] Cassidy H., Poelman R., Knoester M., Van Leer-Buter C.C., Niesters H.G.M. (2018). Enterovirus D68—The New Polio?. Front. Microbiol..

[44] Harvala H., Jasir A., Penttinen P., Celentano L.P., Greco D., Broberg E. (2017). Surveillance and laboratory detection for non-polio enteroviruses in the European Union/European Economic Area. Eurosurveillance.

